# Isolation and Pathogenicity of a Novel Goose Astrovirus from Overfed Adult Landaise Geese in China

**DOI:** 10.3390/v14122806

**Published:** 2022-12-15

**Authors:** Yinchu Zhu, Hongyu Wang, Jionggang Hua, Weicheng Ye, Liu Chen, Zheng Ni, Tao Yun, Jiale Ma, Huochun Yao, Endong Bao, Cun Zhang

**Affiliations:** 1 State Key Laboratory for Managing Biotic and Chemical Threats to the Quality and Safety of Agro-Products, Institute of Animal Husbandry and Veterinary Sciences, Zhejiang Academy of Agricultural Sciences, Hangzhou 310021, China; 2 College of Veterinary Medicine, Nanjing Agricultural University, Nanjing 210095, China

**Keywords:** goose astrovirus, adult goose, isolation, genetic analysis, pathogenicity assessment

## Abstract

Goose astrovirus (GAstV) is an important pathogen causing visceral gout and high mortality in goslings, which has broken out and spread across China. In 2021, a disease characterized by urate deposition on the visceral surface and 30% mortality occurred in commercial adult Landaise geese in Zhejiang Province, China. A systematic study identified an infecting astrovirus, designated ZJCX, that was efficiently isolated from a diseased goose with a chicken hepatocellular carcinoma cell line (LMH). In contrast to other GAstVs originating from goslings, ZJCX caused cytopathogenic effects in LMH cells, and the crystalline arrangement of viral particles was observed through transmission electron microscopy. Indeed, phylogenetic analysis and nucleotide homology comparison revealed that ZJCX isolate belongs to the genotype II cluster of GAstVs and displays 97.8–98.4% identity with other GAstV II strains. However, several specific mutations occurred in the polyprotein and capsid protein regions. Moreover, a pathogenicity assessment of ZJCX with a gosling model was conducted, and typical visceral gout was reproduced and led to 18% mortality. The viral loads of ZJCX in the blood, kidney, and liver were detected with specific primers after inoculation, which demonstrated that the kidney and liver presented viral loads peaking at seven days post-inoculation (dpi). Biochemical parameter examination showed that AST, ALT, γ-GT, UA, and BUN levels were significantly increased by GAstV, whereas body weight was reduced. Overall, this study indicated that the GAstV isolate could infect adult geese, and the results regarding the viral loads and biochemical parameters induced by ZJCX provide insight into GAstV pathogenicity.

## 1. Introduction

The Astroviridae family can be classified into two genera, Mamastrovirus and Avastrovirus, based on the isolation of astroviruses (AstVs) from mammals or avians [1]. Generally, AstVs were reported to be important viral pathogens that mainly cause enteritis diseases in humans and other animals, nephritis in chickens and pigeons, hepatitis in ducklings, and encephalitis in humans, cattle, and sheep [2,3,4,5]. Since 2015, novel goose astroviruses (GAstVs) have emerged and spread rapidly across China [5,6,7,8,9,10]. In contrast to previously reported avian AstVs in terms of disease characteristics, genotypes, and nucleotide homology, GAstV mainly causes gout and death in goslings.

AstVs are nonenveloped, show icosahedral morphology, are approximately 30 nm in diameter, and have positive-sense, single-stranded RNA (+ssRNA) genomes [11]. The length of their genomes ranges from 6.8 to 7.9 kb, consisting of a 5′-untranslated region (UTR), three open reading frames (ORFs), a 3′-UTR, and a poly (A) tail. The first two ORFs, ORF1a and ORF1b, are located towards the 5′ end of the genome and encode nonstructural proteins, including transmembrane (TM) helical motifs, a 3C-like serine protease and an RNA-dependent RNA polymerase (RdRp), which play important roles in AstV transcription, replication, and infectious particle generation [12]. The third ORF is designated ORF2 and encodes the structural protein capsid, which is critical for virion assembly [13].

Currently, GAstV has spread throughout most provinces of China, including Shandong, Henan, Hunan, Anhui, Jiangsu, Zhejiang, Guangdong, and Heilongjiang, causing high morbidity up to 80% and mortality from 10–50% in 5- to 20-day-old goslings, and is characterized by severe urate deposition in the viscera and other interstitial tissues, especially in the heart, liver, and kidney [10,14,15,16]. Infection of goslings results in a low feed conversion rate and growth retardation. It is estimated that the economic losses caused by the epidemic are over 1.5 billion yuan. Thus far, several reports have revealed GAstV in Cherry Valley ducklings, Muscovy ducklings, and other commercial ducks, which broadens the avian species affected by the disease and highlights its significance [17,18,19,20]. However, few studies about the pathogenicity of GAstV have revealed whether, as more GAstV isolates are increasingly identified from different waterfowl species, the pathogenicity and transmission will be increased.

In the present study, an outbreak of gout symptoms in 70-day Landaise geese occurred during the overfeeding period for fat liver in Zhejiang Province in 2021, which led to Landaise geese experiencing continuous mortality rates of approximately 30%. Then, we performed a systematic investigation to identify the causative agent of this disease, and here, we report the isolation and characterization of this pathogen. Indeed, the pathogenicity of this virus was evaluated by experimental infection of goslings. The results showed that GAstV is the aetiological agent of the severe disease. The present data are expected to further our understanding of the pathogenic mechanism and provide theoretical support for the prevention and control of the disease.

## 2. Materials and Methods

### 2.1. Case Review and Sample Processing

In June 2021, an acute outbreak of fatal gout disease occurred in a commercial goose farm in Zhejiang Province, China, which led to the morbidity of approximately 30% (264/880) in 70-day-old Landaise geese. The geese on this farm are used for producing foie gras, which necessitates a high intake of fodder in this period. Tissues from the liver, spleen, and kidney were collected from diseased Landaise geese, randomly selected, and sent to the laboratory for diagnosis.

### 2.2. Bacterial Culture and Viral Nucleic Acid Detection

Laboratory identification of potential bacterial and viral pathogens was performed. The liver and kidney samples collected from dead geese were cultured at 37 °C on tryptic soy broth (TSB) agar plates (BD Science, Sparks, MD, USA) containing 5% sheep blood. Then, tissue samples were pooled and homogenized in phosphate-buffered saline (PBS) and centrifuged at 10,000× *g* for 15 min, and the viral DNA/RNA was extracted from the supernatant using a Takara Virus DNA/RNA Kit (Takara, Dalian, China) in accordance with the manufacturer’s instructions. The nucleic acids were assessed to detect goose parvovirus (GPV), Tembusu virus (TMUV), goose hemorrhagic polyomavirus (GHPV), avian influenza virus (AIV), goose reovirus (GRV), and GAstV with a one-step reverse-transcription (RT)-PCR kit (Vazyme, Nanjing, China) and specific primers.

### 2.3. Virus Isolation and Identification

The supernatants of pooled samples were filtered through a 0.22 µm pore size filter membrane (Merck Millipore, Cork, Ireland), and the filtrate was inoculated into the allantoic cavity of 9-day-old goose embryos (0.2 mL/embryo). The goose embryos used for GAstV isolation were purchased from a healthy breeding farm in Zhejiang, eliminating waterfowl viruses, including GPV, AIV, TMUV, GHPV, GRV, and GAstV. Embryos were incubated at 37 °C and candled daily, and allantoic fluids and embryo bodies were harvested upon embryo death at 24 h and 7 days post-inoculation (dpi) and then preserved at −80 °C. The virus supernatants were inoculated onto chicken hepatocellular carcinoma cell line (LMH) cells, with one hour of adsorption at 37 °C in a 5% CO_2_ atmosphere, and then the medium was replaced with Dulbecco’s modified Eagle’s medium (DMEM/F-12, Basal Media, Shanghai, China) containing 2% FBS and 1% penicillin-streptomycin. The cultures were incubated at 37 °C with 5% CO_2_. In parallel, total RNA was extracted and assayed by RT–PCR with GAstV primers.

### 2.4. Complete Genome Amplification and Sequencing

Viral genomic fragments were amplified by overlap PCR with specific primers designed according to the available GAstV regions in the GenBank database and synthesized (Table S1). The 5′-UTR and 3′-UTR were amplified with primers according to the instructions of the 5′ RACE and 3′ RACE kits (Takara, Dalian, China). In brief, the bands of PCR amplicons showing the target sizes obtained via RT–PCR were purified through a DNA fragment purification kit (Takara, Dalian, China) and ligated into the pEASY-Blunt Zero cloning vector (TransGen, Beijing, China) for further sequencing by Tsingke Biotechnology Co., Ltd. (Beijing, China). Each nucleotide was identified from replicates showing identical genome sequencing results. The nucleotide sequences were assembled using SeqMan (DNASTAR v7.1) and submitted to the GenBank database with the accession numbers.

### 2.5. Sequence Alignments and Phylogenetic Analysis

Sequence similarity searches were conducted with BLAST in the NCBI database (https://blast.ncbi.nlm.nih.gov/Blast.cgi (accessed on 3 December 2022). Reference sequences of other avian AstVs were downloaded from the GenBank database. The nucleotide and deduced amino acid sequences of the isolated strain were compared with the known sequences of avastroviruses (AAstVs) by the MegAlign program v6. To determine the phylogenetic relationships of the newly identified GAstV with the other viruses, complete genome data and three ORFs were further visualized via the neighbor-joining method based on the Kimura 2-parameter model in Molecular Evolutionary Genetics Analysis (MEGA, Auckland, New Zealand, version 6.0), with bootstrap values calculated from 1000 replicates.

### 2.6. Electron Microscopy Observation and Indirect Immunofluorescence Assay (IFA)

LMH cell monolayers were infected with GAstV at an MOI of 0.05 for 48 h. Then, the cells were washed three times with PBS and harvested by centrifugation at 1500 rpm at 4 °C for 5 min. The cell sediment was fixed in 2.5% glutaraldehyde for subsequent transmission electron microscopy (TEM) observation. The samples were dehydrated in propylene oxide for 10 min, embedded in epoxy resin, and examined using a Hitachi H-7650 system (Hitachi, Chiyoda Ward, Tokyo, Japan) according to the manufacturer’s instructions.

IFAs were used to test the expression of viral proteins in infected LMH cells, in which the infected cells were fixed with 4% paraformaldehyde for 10 min, followed by rinsing with PBS. Triton X-100 (0.2% *v*/*v*, Sigma-Aldrich, St. Louis, MO, USA) diluted in PBS was applied to each well for 10 min. A rabbit anti-capsid antiserum against the capsid protein (previously produced in our laboratory) was used as the primary antibody at a 1:400 dilution for 1 h at 37 °C, and the cells were then incubated with a FITC-conjugated secondary antibody for 1 h at 37 °C. Finally, the cellular nuclei were stained with DAPI for 5 min, and the samples were examined by fluorescence microscopy (Zeiss, Oberkochen, Germany).

### 2.7. Pathogenicity Assessment of GAstV in Goslings

To determine the pathogenicity of the newly isolated GAstV, 35 one-day-old healthy Landaise goslings were used for each group (GAstV infected group and PBS control group). The goslings were randomly divided and fed in two separate SPF isolators. All the goslings were purchased from a commercial hatchery in Zhejiang Province, which had not had any instances of GAstV infection before, and previous analysis of viral nucleic acid was performed before experiments. The goslings in the experimental groups were orally challenged with 0.5 mL of GAstV (0.5 × 10 5.66 TCID50/goose) at two days old, the GAstV cultured from LMH cells and titrated with Reed-Muench, and the control group were inoculated with equal doses of PBS. The clinical signs were monitored and recorded every day after infection for 15 days, mainly including behavior, appetite, activity stimulus-response, and mortality rates. On days 1, 3, 5, 7, 10, 13, and 15 dpi, five goslings in each group were randomly selected for serum sample collection and weighing. Indeed, these selected goslings were bled from the jugular vein and euthanized with intravenous administration of pentobarbital sodium (100 mg/kg body weight) for collection of tissue samples, including those for blood, liver, and kidney, at each dpi and stored at −80 °C until detecting and recording the changes in viral load by qRT–PCR. The viral copy numbers were calculated based on the standard curve described in supplement materials (Figure S1). This experiment was designed to investigate the in vivo replication of the virus and its impact on the growth of infected goslings. Tissue samples were prepared as a 10% suspension (*w*/*v*) in PBS and homogenized. Then, the homogenate was centrifuged at 13,000× *g* for 10 min at 4 °C, and 200 μL of the supernatant was used for RNA extraction. RNA was reverse transcribed into cDNA using a HiScript Q RT SuperMix Kit (Vazyme, Nanjing, China) and quantified by quantitative PCR by a thermocycler (ABI7500; Life Technologies, Carlsbad, CA, USA).

### 2.8. Histopathological Examination

The goslings that died or were sacrificed at the corresponding time after inoculation were autopsied. The liver and kidney samples were collected for further histopathological analysis. Tissue samples were placed in 4% (*v*/*v*) paraformaldehyde fixative solution at room temperature and then routinely processed, dehydrated with alcohols, clarified in xylene, and embedded in paraffin. The paraffin block tissues were serially cut into 5 μm sections and mounted on glass slides. Finally, the sections were stained with hematoxylin-eosin (HE) and examined with light microscopy to evaluate pathological tissue damage. Gomori methenamine silver (GMS; Solarbio life sciences, Beijing, China) was used to stain the urate according to the procedure. The tissue had been fixed in absolute ethanol overnight, and the Gomori methenamine silver could stain the crystal of urate with black color.

### 2.9. Serum Biochemical Parameter Analysis

The serum samples collected at each corresponding time after centrifugation (10 min, 5000 rpm) were stored at −20 °C, which were used to detect and record the changes in biochemical parameters. The following 11 biochemistry parameters were measured by an automatic serum chemistry analyzer (Celercare V5; MNCHIP, Tianjin, China) according to the manufacturer’s protocols: total bilirubin (TBIL), total protein (TPRO), alanine aminotransferase (ALT), aspartate aminotransferase (AST), globulin (GLOB), albumin (ALB), albumin:globulin ratio (A/G), creatinine (CRE), blood urea nitrogen (BUN), γ-glutamyltransferase (γ-GT), and glucose (GLU). The level of uric acid in serum was measured by a commercial kit (Nanjing Jiancheng Bioengineering Institute, Nanjing, China). The damage caused by GAstV could lead to changes in biochemical parameters.

### 2.10. Statistical Analysis

The data from all the experiments were plotted using GraphPad Prism (v.7) software for statistical analyses. The differences between the control group and experimental group were analyzed by an Unpaired two-tailed Student’s *t*-test, and variables presented as the mean ± standard deviation (SD) were used for evaluation. For all tests, a *p*-value < 0.05 was considered statistically significant (* *p* < 0.05, ** *p* < 0.01) compared with the control group.

### 2.11. Ethics Statement

All animal experiments were conducted in accordance with the Guidelines for Experimental Animals of the Ministry of Science and Technology (Beijing, China) and were approved by the Institutional Animal Care and Use Committee (IACUC) of Zhejiang Academy of Agricultural Sciences (protocol code 2022ZAASLA42). All goslings used in the experiment were euthanized by intravenous injection of sodium pentobarbital (100 mg/kg body weight).

## 3. Results

### 3.1. Identification and Isolation of GAstV from Samples

**Figure 1:**
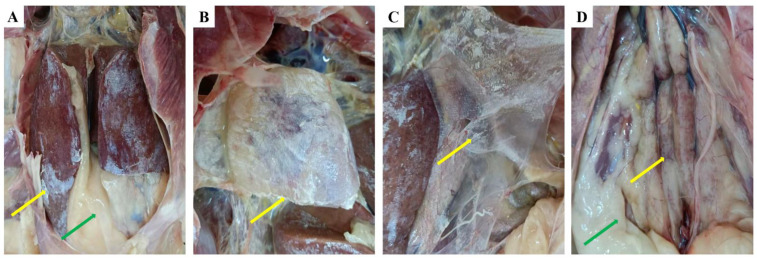
Postmortem lesions of diseased Landaise geese from the commercial farm. (**A**) Urate deposition on the surface of the liver and excessive fat accumulation in the abdominal cavity, (**B**) urate deposition in the heart and pericardium, (**C**) urate deposition in the peritoneum, (**D**) fat accumulation around the kidney and severe nephritis with swelling. The yellow arrow indicates urate deposition, and the green arrow indicates fat accumulation.

In this field case, the diseased geese displayed signs of loss of appetite, depression, and death. The characteristics of anatomically displayed common features were visceral urate deposition on the surface of the liver and pericardium, renal hemorrhage enlargement and urate deposits in the ureter and serosa, and excessive fat accumulation in the abdominal cavity (Figure 1).

To investigate the causative agents, samples were collected for viral DNA/RNA extraction and were confirmed to be positive only for GAstV; the other waterfowl viruses displayed negative results by RT–PCR analysis. Moreover, no bacteria were detected in the diseased tissue. Notably, GAstV has been previously reported to infect only goslings and ducklings younger than 20 days, so this is the first time that a GAstV outbreak has been reported in adult geese.

**Figure 2:**
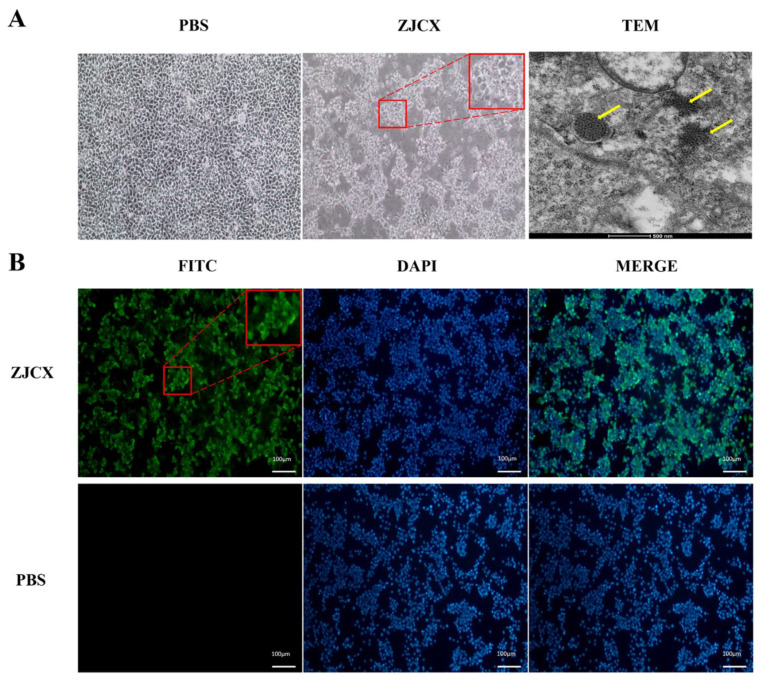
Isolation of GAstV in LMH cells and identification of the GAstV ZJCX isolate by indirect immunofluorescence assay. (**A**) LMH cells with PBS inoculation as a negative control. The ZJCX inoculation could cause CPE in LMH cells (the red magnification was 2-fold, X2) and transmission electron micrograph of ZJCX in cells displaying a crystalline arrangement (yellow arrow). (**B**) LMH cells infected with ZJCX were reacted with rabbit serum against the capsid protein at 48 hpi, and a secondary antibody labeled with FITC displayed a green signal (the red magnification was 2-fold, X2). PBS as the negative group only displayed a blue signal, which was stained with DAPI for the cell nucleus.

In addition, the isolation of GAstV was initiated by inoculating homogenates of the pooled liver and kidney specimens from diseased geese into the chicken liver cell line LMH. The virus caused the cytopathogenic effect (CPE) to appear after 72 h of inoculation, while the cells in the PBS control group were still smooth and intensive (Figure 2A). After three passages, the infected LMH cells were collected, discarded from the culture, and fixed with 2.5% glutaraldehyde. Subsequently, a transmission electron microscope was employed to identify the virus. A large number of virus particles were observed, which showed a crystalline arrangement and uniform spherical particles with diameters of 30 nm in LMH cells (Figure 2A). Thus, this is the newly isolated GAstV designated ZJCX. The results of IFA demonstrated that specific green fluorescence signals could be observed in the cytoplasm of ZJCX-infected LMH cells, and the cell nucleus stained with DAPI was blue (Figure 2B). All these results clearly demonstrated that GAstV could efficiently proliferate in LMH cells.

To further confirm the isolation of this virus, ZJCX was inoculated into 9-day-old healthy goose embryos. After five passages, the isolate caused 20–50% mortality of the embryos by 6 dpi. The dead embryo bodies exhibited severe subcutaneous hemorrhage and edema (Figure S2). Eventually, the newly isolated GAstV was consistently detected in the allantoic fluids with specific one-step RT–PCR.

### 3.2. Complete Genome Sequence Analysis

To reveal the characteristics of this isolated strain and determine the relationships between it and other GAstVs circulating in goslings, the full-length genome of the isolate ZJCX was sequenced with overlapping RT–PCR and RACE strategies. Sequence analysis showed that ZJCX was 7182 nt in length, with an 18 nt 5′UTR, a 3255 nt ORF1a, a 1551 nt ORF1b, a 2115 nt ORF2, a 206 nt 3′UTR, and a poly-A tail (29 nt), and this sequence was submitted to NCBI (GenBank accession number ON745304). The three coding regions were predicted to encode a polypeptide of 1084 aa (ORF1a), including a trypsin-like peptidase domain, a nuclear localization signal (KKKGKTK), and four predicted transmembrane domains: An RdRp of 516 aa (ORF1b) and a capsid protein of 704 aa (ORF2). Indeed, a highly conserved ribosome translocation frameshift signal, AAAAAAC, was observed between ORF 1a and ORF 1b overlapping regions and a downstream stem-loop structure (3264–3270 nt), which was predicted by RNA folding analysis. The complete genome sequences of GAstV strains were input into RDP4 for recombinant analysis to explore the potential evolutionary process of GAstV, and the results demonstrated that no recombinant event occurred in the ZJCX genomic sequence.

### 3.3. Genetic and Evolutionary Analysis of AstVs

To better understand the evolutionary relationships between GAstV ZJCX and other AAstV members, the complete genome sequence alignments were compared with the representative reference AAstV strains. The genomic data of AAstV strains were downloaded from the GenBank database for comparative genomic and phylogenetic analysis. The complete genome sequence of GAstV ZJCX shared the highest similarity and identity with previously reported GAstV isolates (representatives GD, AH02, ZJLD, etc., in Table 1), excluding the AHDY, FLX, TZ03, and ZJC14 strains (50.3–57.8%). Moreover, this new strain ZJCX shared 32.0 to 61.5% identity with other avian species members. Further comprehensive analysis of the nucleotide and amino acid sequences was performed with other known avian AstVs, as determined by BLAST, and is summarized in Table 1. At the nucleotide level, the ORF1a, ORF1b, and ORF2 gene sequences of ZJCX presented homology ranging from 28% to 57.7%, 50.4–67.6%, and 23% to 55.4% (ANV II to TAstV II) with those of other AstVs, respectively. At the amino acid level, the homology was 73.1% to 82.9%, 83.1% to 97.2%, 64.1% to 66.9%, and 51.3% to 57.5%, respectively.

**Figure 3:**
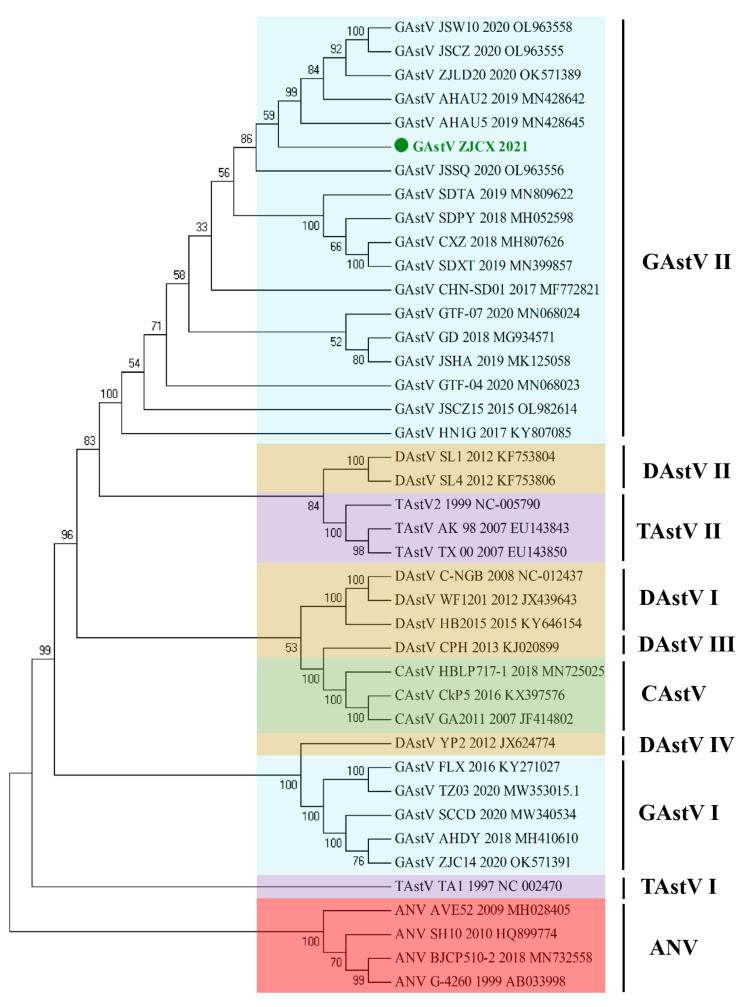
Phylogenetic analysis of whole genomes between ZJCX and AAstV reference strains. Multiple alignments of complete genomes of other GAstVs and representative strains of AAstVs from the GenBank database was performed. Phylogenetic analysis based on the nucleotide sequences was constructed with MEGA 6.0 software using the neighbor-joining method with 1000 bootstrap replicates.

**Figure 4:**
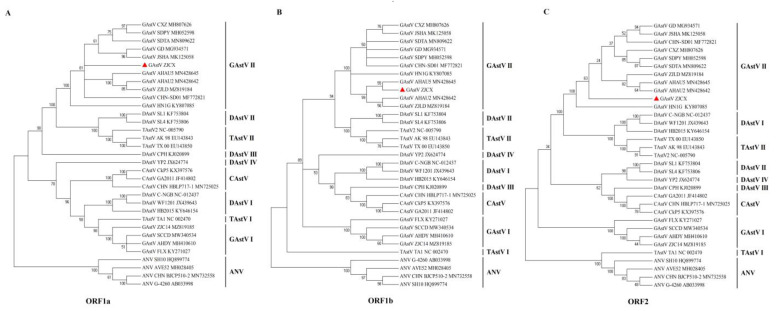
Phylogenetic analysis based on the amino acid sequence of ORFs between ZJCX and AAstV reference strains. Phylogenetic analysis of astrovirus ORF1a (**A**), ORF1b (**B**), and ORF2 (**C**) sequences using MEGA 6.0 with the neighbor-joining method with 1000 bootstrap replicates and the composite likelihood model. The isolate ZJCX is indicated in red.

Additionally, the phylogenetic tree of the full-length sequences was constructed with MEGA 6.0 software and showed that strain ZJCX belonged to the same branch as ZJLD, AHAU2, JSSQ, GD, and JSHA, which are closely related to DAstV-II and TAstV-II, designated the GAstV II group (Figure 3). ZJCX was considerably distant from the GAstV-I strains, including ZJC14, AHDY, FLX, SCCD, and TZ03. The phylogenetic analysis of individual genes, including ORF1a, ORF1b, and ORF2, revealed a grouping structure close to and matching that of the AstV whole genomes (Figure 4). In addition, another analysis based on the genomes of most of the GAstV II isolates suggested that ZJCX was close to the AH02 strain in terms of phylogenetic relationships (Figure S3).

The alignments of the deduced amino acid sequence of strain ZJCX with that of other GAstV II strains were determined according to the amino acid sequence variations in the ORFs. The results demonstrated that the polyprotein ORF1a and capsid protein were potential variable regions with mutations occurring in different clinical isolates (Table S2). For example, there were T188A and S265T mutations in the transmembrane domain, an A527V mutation occurred in the serine protease domain, and six mutations (A890V, Q910P, E911K, V912G, D918G, and A927V) occurred in the nuclear localization signal region (NLS), and ZNF of the ORF1a protein was specific to the other GAstV II strains. Notably, the characteristic motifs GNSG and KKKGKTK in the serine protease domain and NLS remained the same in ZJCX. In the ORF1b protein as the predicted RdRp, only three mutations occurred, which showed that ZJCX ORF1b produces a conserved protein. The ORF2 capsid protein is the core element of the antigen, leading to recombination and mutation, such as Q229P in the inner core region and I498L, S586T, N587D, E610G, E634D, and A695T in the spike region (P2).

### 3.4. Replication Kinetics and Histopathology of GAstV-Infected Goslings

Healthy goslings were infected with strain ZJCX to determine its pathogenicity. Some infected goslings exhibited signs of depression at 3 dpi, and nine deaths occurred from 5–11 dpi, resulting in a mortality rate of 18% (9/50). Meanwhile, no significant clinical signs or death were observed in the PBS control group. In this animal experiment, the weight and viral replication of each gosling were determined at 1, 3, 5, 7, 10, 13, and 15 dpi. The results showed that the infected group had a remarkably lower weight than the uninfected group from 7 dpi until the end of the experiment, which indicated that GAstV significantly influenced the growth of goslings.

**Figure 5:**
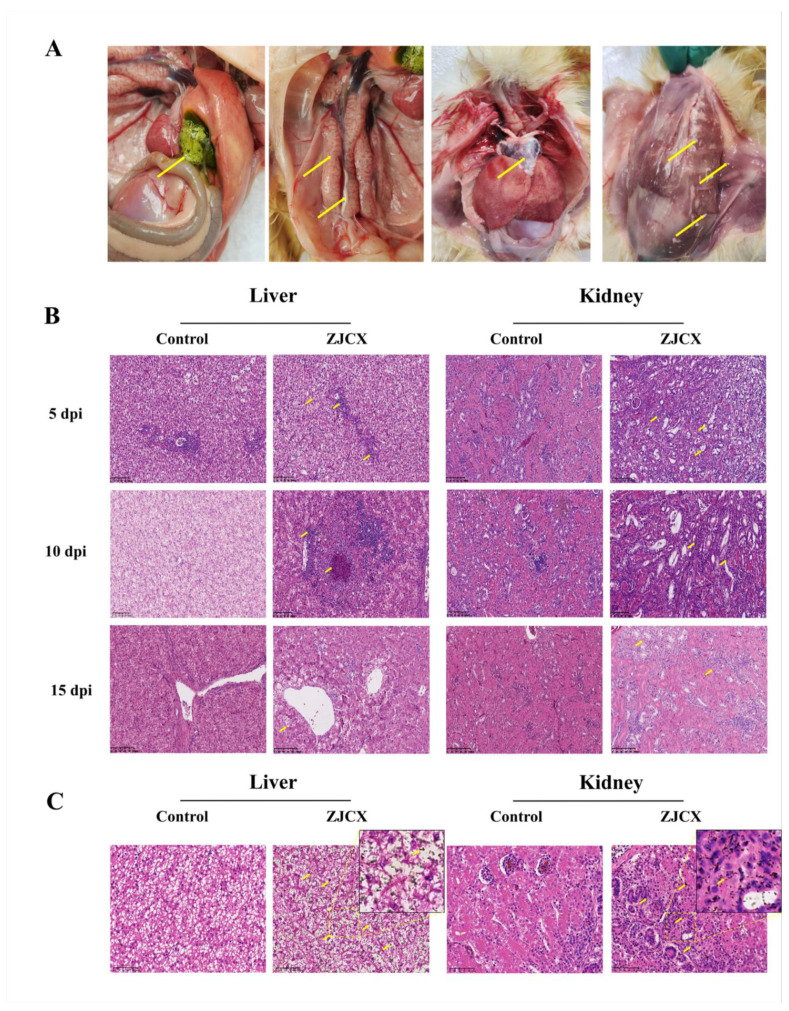
Postmortem and histopathology lesions of tissues from goslings infected with GAstV. The dead goslings at 7 dpi displayed significant visceral gout with urate deposition (yellow arrow). Urate deposits in the gallbladder, ureters, peritoneum, pericardium, severe hemorrhage, and swellings of kidneys (**A**). HE-stained liver and kidney section with experiment goslings 5, 10, and 15 dpi showed necrosis, diffuse hemorrhage, renal tubular epithelial cell degeneration, and exfoliation. The histopathology change was shown with a yellow arrow. Magnification, ×200 (**B**). GMS stain of liver and kidney tissues from infected goslings 5 dpi showed urate with black color (yellow arrow) and deposits in the tissue (**C**).

At the autopsy of these dead goslings, a small number of urate deposits were observed on the surface of the heart and accumulated in the gallbladder, where they were the most conspicuous. Moreover, white substance-filled ureters, severe renal hemorrhage, and swelling were also observed (Figure 5A). The other infected goslings who survived showed hemorrhage and renal enlargement without significant symptoms of gout. In addition, histopathological analysis with HE staining of pathological sections was used to assess the extent of histopathological damage. There was inflammatory cell infiltration around the central vein of the liver. A large amount of renal tubular epithelial cell necrosis and nuclear pyknosis or rupture (Figure 5B). The GMS staining of the liver and kidney from goslings with GAstV infection revealed localization of urate deposits inside the tissue, displaying an amount of black urate. On the contrary, no obvious staining of urate was observed in the tissues from the control group goslings (Figure 5C).

**Figure 6:**
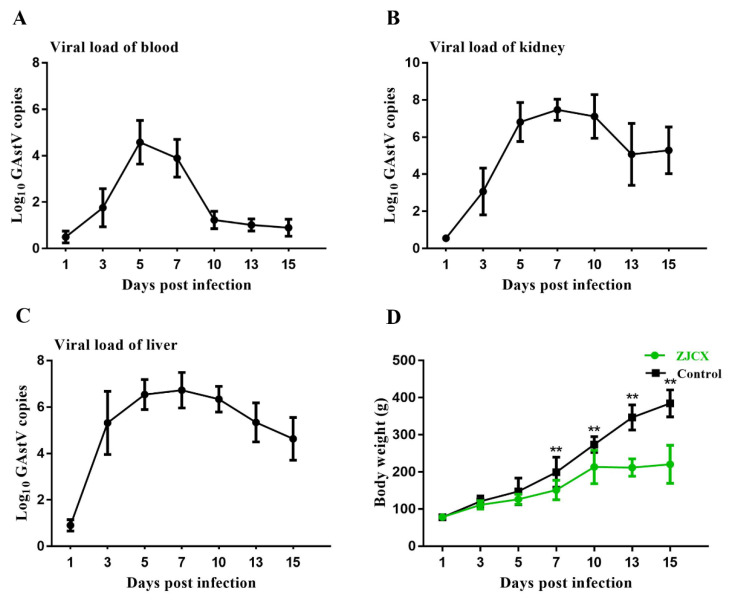
Dynamic replication kinetics of ZJCX and the body weight changes of infected goslings. Total RNA was extracted from the tissues and measured by qRT–PCR. (**A**) The viral copy numbers in the blood of the infection group. The viral load in the kidney (**B**) and liver (**C**) of the infected group. (**D**) Changes in weight of goslings after infection compared with that of goslings in the PBS control group. Values are expressed as mean ± SD, ** *p* < 0.01.

Furthermore, to explore the viral replication and distribution within goslings, blood, liver, and kidney samples were obtained at specific times for viral RNA detection. The detection results of the GAstV copy number in the samples are shown in Figure 6. Viral RNA in the tissue of the infected group could be detected at 3 dpi, and the virus reached peak replication between 5 and 7 dpi. Notably, viral copy numbers of ZJCX in the liver were higher than those in the kidneys early in the infection course, reaching 10^5^ copies in the liver at 3 dpi, while the virus load was only 10^3^ copies in the kidney. Conversely, the peak viral load in the kidney was higher than that in the liver after 5 dpi. In blood, the virus was detected at only 10^2^ copies at 3 dpi and peaked at 10^4^ copies. Then, the viral load in blood decreased quickly over time, which showed a different trend than that in other collected tissue samples. Moreover, no viral RNA was positively detected in the negative control group.

### 3.5. Elevated Biochemical Parameters Induced by GAstV Infection

**Figure 7:**
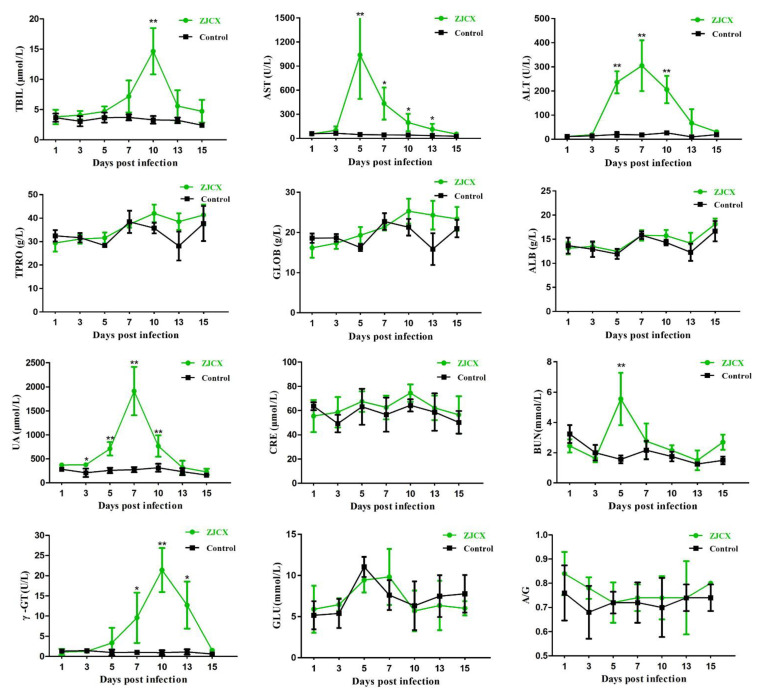
Abnormalities in biochemical parameters of hepatic and renal function. Dynamic changes in 12 biochemical indexes in the serum of the experimental goslings were compared between the infected and control groups at different days post-infection. TBIL, AST, ALT, UA, BUN, and γ-GT showed significant increases caused by ZJCX. * *p* < 0.05; ** *p* < 0.01.

Viral infection causing potential damage in the host could be indicated through biochemical detection. Significant differences were found in the biochemical parameters of liver enzymes between the infection and control groups, which suggested that the activities of the liver enzymes ALT, AST, and γ-GT and TBIL levels were conspicuously higher in the GAstV-infected group. The risk of renal function damage implied that the parameters UA and BUN were obviously higher in the GAstV-infected group. The results were consistent with previous reports on humans and other animals with gout disease. Briefly, the dynamic changes in biochemical parameters caused by GAstV in different groups of goslings are presented in Figure 7. The analysis demonstrated that changes in diverse biochemical parameters occurred at different dpi; AST and BUN peaked at 5 dpi, ALT and UA at 7 dpi, and TBIL and γ-GT at 10 dpi. The changes in the above biochemical parameters persisted for approximately one week and declined quickly with time after reaching peak levels, returning to normal levels at 15 dpi. In addition, the other six biochemical indexes were detected, but none of them displayed any difference between the experimental group and the control group. AST, ALT, γ-GT, UA, and UN in serum from GAstV-infected goslings were significantly higher than those in the PBS control group, which accurately confirmed that this virus causes renal and hepatic injuries to disrupt metabolic pathways, providing valuable guidance to understand liver and kidney trauma.

## 4. Discussion

Several studies have reported the prevalence and isolation of GAstV causing visceral gout and high mortality among domestic goslings, which has led to an enormous economic loss to the goose industry in China. Indeed, GAstV infection occurred in different species of ducklings, which indicated the potential cross-species transmission of AstV between domestic waterfowl [17,18,19,20]. However, the pathogenicity of GAstV among geese remains poorly understood, while more outbreak cases have been reported in various areas, and efficient diagnostic assays have been constructed [14,21,22,23]. Generally, GAstV infection occurs within 5–20 days of age, after which goslings recover with growth according to the epidemiological investigation, and there was a study estimated that GAstV failed to cause visceral urate deposition or death in goslings over 25 days of age which indicated the pathogenicity closely related to the host age [24]. However, in May 2021, a disease characterized by urate deposition in the viscera, kidney tubules, and ureters occurred in a commercial Landaise goose farm during the overfeeding period at approximately 70 days of age in Zhejiang Province and finally resulted in 40% death. Based on clinical signs, the causative agent of this outbreak disease was subsequently identified as a GAstV strain, and other potential viruses, such as AIV, GPV, and DTMUV, were excluded by RT–PCR assay. Hence, we integrated a variety of assays to understand the genetic and pathogenic characteristics of the new GAstV strain ZJCX.

In this study, the causative agent GAstV ZJCX was isolated, and it could propagate well both in goose embryos and LMH cells. The IFA assay indicated that the capsid proteins of ZJCX were similar to those of GAstV from other goslings, such as ZJLD and JSW10. However, ZJCX could easily produce cytopathic effects in each passaging of infected LMH cells, which is different from the strain GD [25]. A detailed analysis and experiment should be carried out to explore the potential reason. In addition, the comparison and phylogenetic analysis of AstV based on the full-length genome sequences revealed that the ZJCX strain belonged to the GAstV II group, with high similarity in sequence identity and close evolutionary relationships with the representative strain. Some particular amino acid mutations occurred in the three predicted proteins, which may result in the various characteristics of ZJCX and GD strains in producing CPE and infected adult geese. Further experiments are required to test this prediction. Notably, strain ZJCX displayed the highest similarity in genome sequence with AH02 and was assigned to the same subgroup based on evolutionary relationships of GAstV II strains, whereas ZJCX was located far from ZJLD and XSSH in the phylogenetic tree, although they all originated from Zhejiang Province. This result may indicate that multiple goose production systems allow goslings and geese to be traded throughout the country, and freight vehicles, contaminated feed, and infected hosts may be carriers mediating pathogen transport, leading to widespread virus transmission. The infection and control gosling groups placed in the same incubator led to the infection of the control group. These findings revealed that the GAstV strain had a capacity for horizontal transmission and caused pathogenesis in geese of different ages [24].

Indeed, a systematic investigation of the pathogenicity of GAstV in the gosling model was performed. In this experiment, strain ZJCX reproduced the clinical signs of visceral gout and death, while mortality was only 20% in the laboratory. In the clinical case, overfeeding may attack the liver, which may promote GAstV infection in adult geese. The kidney and liver are the main functional organs responsible for the visceral gout process [24,26], so the replication dynamics of ZJCX in vivo, including in the blood, kidney, and liver, were assessed, and the results suggested that viral loads could be obviously detected in these collected samples at 3 dpi. Moreover, viral load in the liver and kidney reached a peak at 7 dpi, and that in blood at 5 dpi; the loads in the liver and kidney were also significantly, approximately 100 times higher than those in blood, and the virus can replicate efficiently in liver and kidney, impairing kidney and liver function, which indicates an increased potential risk for producing urate crystals. In addition, GAstV was continuously detected in the liver and kidney throughout the experiment with high copy numbers, but the virus was difficult to detect in blood after 7 dpi due to the antiviral effect of the immune system. Thus, the liver and kidney are the best target samples for GAstV detection in clinical aetiological diagnosis. For viral shedding, there was study revealed that the virus could be first detected in cloacal swabs in 2 dpi, and lots of shedding viruses were similar to the trend of viral loads in tissue, reached a peak value at about 7 dpi, followed by the decline with the time [24]. Virals released from the cloaca contaminate feed and drinking water, causing more widespread infections, indicating that it is urgent to take effective measures to control the spread of disease [27]. Besides, there was research about the effect of age at GAstV infection; GAstV infection caused damage to the immune organs, leading to multi-organ dysfunction and inducing replication of the pathogen [24]. The immune organs of the goslings were un-mature compared with adult geese, which would lead to high morbidity and mortality rate of natural outbreaks in goslings, and goslings may lead to death with urate deposition on the surface of organs, but without displaying other significant symptoms. The pathogenesis of the age effect also needed to be elucidated. In consideration of some infected goslings survived during the experimental observation, and the infection dose of GAstV, protein level in fodder, environmental temperature, and humidity during breeding also need to be further elucidated as factors that promote gout disease.

Biochemical parameters for abnormalities in hepatic and renal function are critical for the pathogenesis of GAstV [28]. Organ damage causes disorders in the enzyme system, leading to the release of enzymes from cells into the blood. AST is present in the cytoplasm of the liver, ALT is located in the mitochondria of hepatocytes, γ-GT is present in blood from the liver, and TBIL is mainly produced in the liver [29,30]. They are specific markers of liver injury because the liver is one of the main metabolic organs; if the liver cells are damaged, or the biliary tract is blocked, hepatocyte degeneration or permeability increases, and enzymes leak into the blood, resulting in high concentrations [30,31,32]. Therefore, the sharply elevated serum levels of AST, ALT, and γ-GT after 3 dpi in this study indicate a reduction in liver function, while the high level lasted for a short duration. Kidney function-related indicators such as UA and BUN are closely associated with purine metabolism, and elevated concentrations in serum revealed gout and nephrolithiasis caused by renal damage [33,34]. AST, ALT, γ-GT, UA, and UN in serum from GAstV-infected goslings were significantly higher than those in the PBS control group, which accurately confirmed that this virus causes renal and hepatic injuries to disrupt metabolic pathways, providing valuable guidance to understand liver and kidney trauma. The level of enzymes is closely related to dynamic changes in viral loads, and the viral load reached a high level, leading to a high concentration of these biochemical enzymes and decreasing with time. The self-repair and immune elimination functions of the host in the later infection period may be the possible reason for the reduction in the levels of the enzymes.

In this study, GAstV infection leads to severe liver and renal damage, which would promote uric acid synthesis and impeded uric acid excretion, and increase serum uric acid. The rate of kidney urate formation is greater than the excretory capacity of the urinary organs and then causes gout. Uric acid accumulates in the blood and can be transferred to any of the organs of the body through blood circulation leading to urate deposits such as in the heart, liver, and kidney, which have more obvious gout lesions. Furthermore, histopathological observations displayed renal tubular epithelial cell degeneration and necrosis in infected goslings in consistency with other previous studies. The histopathological examination supported the process by which invasion of GAstV damaged the tissue and cells, resulting in leakage of metabolism-related enzymes. However, the specific mechanism underlying this effect is unclear. Notably, poultry, unlike mammalian species, lack arginase, leading to ammonia accumulation that cannot be resolved into urea instead of purine, hypoxanthine, and xanthine, which would be utilized as raw materials for forming urate [35]. Therefore, the abnormal hepatic and renal function caused by GAstV promotes urate formation and destroys the excretory capacity, resulting in urate deposits on visceral surfaces. A report also revealed that AstVs could increase the permeability of epithelial cells, which may contribute to gout disease [36,37].

GAstV infection can reduce feed intake and change the efficiency of the feed conversion ratio, thus leading to growth suppression. Although the condition of the main organ in surviving goslings will recuperate, the body weight of GAstV-infected goslings was reduced by approximately 40% because of the decreased ingestion and conversion capability, which led to a tremendous economic loss in the goose industry.

## 5. Conclusions

In summary, this is the first demonstration of GAstV causing gout disease and death in adult geese, which portends the possibility of this virus causing pathogenesis in geese of all ages. In contrast to GAstV isolates from goslings described in previous reports, the ZJCX strain could efficiently proliferate in LMH cells and cause CPE. Whether or not the variability of the genomic sequence results in different characteristics needs to be verified. The animal experiment successfully reproduced the symptoms of urate deposition and death in the gosling model. In addition, this study determined the replication dynamics in vivo and the accurate values of biochemical parameters induced by GAstV, which provides useful information for further analysis of the pathogenic mechanism. Indeed, further detailed research is urgently needed to understand the virulence of the emerging virus and to develop effective measures/vaccines against GAstV that promote the development of the goose industry in China.

## Figures and Tables

**Table 1:** Comparison of amino acid sequence identities between ZJCX and other Avastrovirus genera.

Species	Virus Strains	GeneBank No.	Identity (%)						
			Genome	ORF1a		ORF1b		ORF2	
			nt	nt	aa	nt	aa	nt	aa
Avastrovirus 1	TAstV –I TA1	NC_002470	38.9	26.0	36.6	57.3	53.5	32.4	38.9
Avastrovirus 2	ANV-I G-4260	AB033998	32.9	21.4	28.2	56.5	50.6	18.8	23.1
	ANV-II AVE52	MH028405	32.0	19.0	28.0	53.8	50.4	18.6	23.0
Avastrovirus 3	TAstV-II TX/00	EU143850	61.3	62.4	57.7	67.6	67.6	59.3	55.4
	DAstV-I C-NGB	NC-012437	60.4	59.5	47.4	65.1	63.8	58.3	55.1
Unassigned	DAstV-II SL4	KF753806	61.5	63.2	56.2	67.1	67.2	28.7	35.9
	DAstV-III CPH	KJ020899	59.2	59.9	48.8	65.4	63.2	31.2	34.7
	DAstV-IV YP2	JX624774	58.2	57.7	40.7	64.5	60.7	29.1	32.4
	CAstV CkP5	KX397576	58.7	58.5	47.7	65.1	63.6	29.0	34.2
	GAstV-I FLX	KY271027	50.3	40.8	45.2	64.9	59.5	27.5	37.4
	GAstV-I AHDY	MH410610	57.7	57.0	47.2	64.6	61.0	52.1	40.1
	GAstV-I TZ03	MW340534	57.4	56.8	47.1	64.5	60.8	52.5	40.8
	GAstV-I SCCD	MW353015.1	57.8	57.0	47.2	64.6	60.6	54.4	40.3
	GAstV-I ZJC14	OK571391	50.4	40.9	45.2	65.5	59.7	53.5	37.8
	GAstV-II GD	MG934571	97.9	97.7	98.7	98.2	99.0	97.9	98.4
	GAstV-II AH02	MN307116.1	98.74	98.5	98.8	98.6	99.0	99.0	98.9
	GAstV-II JSHA	MK125058.1	97.9	97.4	98.7	98.5	99.0	98.2	98.4
	GAstV-II ZJLD	OK571389	98.3	97.8	98.8	98.9	99.0	98.1	97.9
	GAstV-II HNXX-6	MW592379.1	98.2	97.8	98.9	99.1	99.0	98.0	98.0
	GAstV-II JX01	MZ576222.1	98.1	97.7	99.2	99.0	99.0	97.9	98.2

## Data Availability

The data that support the findings of this study are available from public database GenBank, and the accession number of ZJCX is ON745304.

## Supplementary Materials

The following supporting information can be downloaded at: https://www.mdpi.com/article/10.3390/v14122806/s1, Table S1: Primers used for the amplification of full-length genomes of the GAstV strain ZJCX; Table S2: Analysis of the main functional amino acid sites between ZJCX and other GAstV II ORFs; Figure S1: Establishment of a standard curve for the Duplex RT-qPCR assay; Figure S2: Isolation and identification of GAstV in goose embryos; Figure S3: Phylogenetic tree of most GAstV II strains in China based on whole genome sequences.
