# Supplementary material for: Isolation and Pathogenicity of a Novel Goose Astrovirus from Overfed Adult Landaise Geese in China
Source: Viruses. 2022 Dec 15;14(12):2806. doi: 10.3390/v14122806 (PMC9784181; doi:10.3390/v14122806)
Supplement: Supplementary file 1 [file viruses-14-02806-s001.zip › viruses-2060745-supplementary.pdf]

**Table S1.** Primers used for the amplification of full-length genomes of the GAstV strain ZJCX.

| Primers | Sequences (5′-3′) | Amplicon size (bp) | Purpose |
|---|---|---|---|
| GAstV 1F | CCGAAACAGCGATATGGCGGCCGGTG | 2266 | Genome sequencing |
| GAstV 2246R | GTCCTCTCGCAATTCCTCAA | | |
| GAstV 1976F | CTGGCTGGTGTATTATTGATG | 1198 | Genome sequencing |
| GAstV 3154R | GACCATCAAACAATGAGTGCAT | | |
| GAstV 3049F | GAGCAGCGCAAAAGGATCTG | 1498 | Genome sequencing |
| GAstV 4529R | TAAGACCACAGAAAGTCATA | | |
| GAstV 4440F | GACACGAATGTCGTCATAG | 2698 | Genome sequencing |
| GAstV 7119R | CTTAAAAATCACATTTGATTC | | |
| GAstV 6795F | GACTTCCACCTAGCAGTCTC | NA | 3′RACE |
| GAstV 6838F | AAGAAGAGGCTGAGTACTGGA | | nest PCR |
| GAstV 412R | GTTGGACAAGAGCTCCACATCG | NA | 5′RACE |
| GAstV 204R | GCATTACACTTCATGAGAGCA | | nest PCR |
| GAstV II-F | GAGCAGGACCAGAATGAGAAA | | qRT-PCR |
| GAstV II-R | CACCACCAATGAGCCTAGATAC | | |
| β-actin-F | ACATCAGGAAGGACCTGTACG | | qRT-PCR |
| β-actin-R | GGGGCGATGATCTTGATC | | |
| OASL-F | CAGCGTGTGGTGGTTCTC | | qRT-PCR |
| OASL-R | AACCAGACGATGACATACAC | | |
| TLR-3-F | CTCTCTGGAAAAAAATAAGCAATTTG | | qRT-PCR |
| TLR-3-R | CTCAAGCAAAGTGCATGATTGC | | |
| TLR-7-F | CACAGAAAAATGGTACCTC | | qRT-PCR |
| TLR-7-R | TACATCGCAGGGTAAACT | | |
| IFN-$\alpha$-F | CAGCACCACATCCACCAC | | qRT-PCR |
| IFN-$\alpha$-R | TACTTGTTGATGCCGAGGT | | |
| Mx2-F | TTCACAGCAATGGAAAGGGA | | qRT-PCR |
| Mx2-R | ATTAGTGTCGGGTCTGGGA | | |
| IL-6-F | AAGTTGAGTCGCTGTGCT | | qRT-PCR |
| IL-6-R | GCTTTGTGAGGAGGGATT | | |
| IL-8-F | GCTGTCCTGGCTCTTCTCCTGATT | | qRT-PCR |
| IL-8-R | GGGTCCAAGCACACCTCTCTGTTG | | |
| IL-10-F | TGCCTCCACTTGTCTGACC | | qRT-PCR |
| IL-10-R | TCCTCCATGTAGAACCGCATC | | |
| IFITM1-F | ATCAAGGCCCGAGATAGGA | | qRT-PCR |
| IFITM1-R | AGAGTATCACCAGGACCAGAA | | |
| TRIM25-F | CTGGATTTCAACACAGCTCATAAC | | qRT-PCR |
| TRIM25-R | CCCAGCACTTGGGAACAATA | | |

**Table S2.** Analysis of the main functional amino acid sites between ZJCX and other GAstV II ORFs.

| Protein | Position | Isolates | | | | | | | |
|---|---|---|---|---|---|---|---|---|---|
| | | ZJCX | AH02 | GD | JSCZ15 | JSW10 | ZJLD | G576 | Others |
| ORF1a | 175 | M | I | M | I | M | M | M | M/I |
| | 188 | A | T | T | T | T | T | T | T |
| | 265 | T | T | S | S | S | S | S | S |
| | 355 | I | I | V | V | V | I | V | V/I |
| | 428 | N | N | T | T | N | N | T | N/T |
| | 520 | T | T | A | A | T | T | A | T/A |
| | 527 | V | A | A | A | A | A | A | A |
| | 528 | T | T | I | I | T | T | I | T/I |
| | 535 | R | R | S | S | R | R | S | R/S |
| | 555 | V | V | V | V | I | I | V | I/V |
| | 890 | V | A | A | A | A | A | A | V/A |
| | 910 | P | Q | Q | Q | Q | Q | Q | Q |
| | 911 | K | E | E | E | E | E | E | E |
| | 912 | G | V | V | V | V | V | V | V |
| | 918 | G | D | D | D | D | D | D | D |
| | 927 | V | A | A | A | A | A | A | A |
| ORF1b | 30 | T | I | I | I | I | I | I | I |
| | 167 | R | K | K | K | K | K | K | K/R |
| | 415 | R | K | K | K | K | K | K | K |
| ORF2 | 229 | P | Q | Q | Q | P | P | P | P/Q |
| | 289 | A | A | A | T | T | A | T | A/T |
| | 456 | E | E | E | E | D | D | D | D/E |
| | 464 | N | N | A | A | N | N | N | A/N |
| | 498 | L | L | I | I | I | I | I | L/I |
| | 540 | L | L | L | L | Q | Q | Q | L/Q |
| | 586 | T | S | S | T | S | S | S | T/S |
| | 587 | D | N | N | N | N | N | N | N |
| | 610 | G | E | E | E | E | E | D | E/D |
| | 614 | A | A | A | A | T | A | N | A/N/S/T |
| | 634 | D | E | E | D | E | E | E | D/E |
| | 695 | T | A | A | A | A | A | A | A |

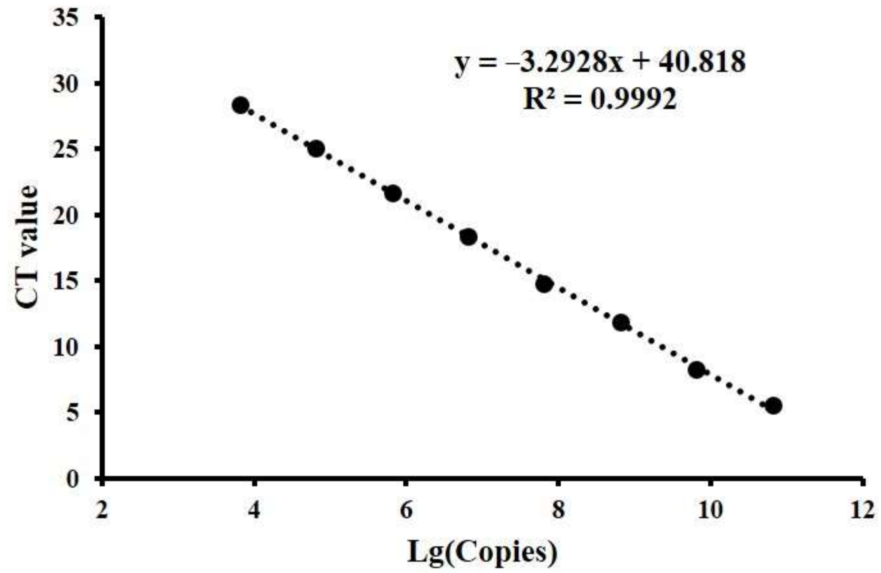


**Figure S1.** Establishment of a standard curve for the Duplex RT-qPCR assay. The RdRp gene fragment was amplified by PCR using GAstV-II cDNA template. The PCR product was purified and linked to the pEASY–T5 vector. DNA concentrations were determined and the copy number per μL was calculated according to the formula: $6.02 \times 10^{23}$ copies/mol × Concentration/Average molecular weight. The recombinant standard plasmid with $6.49 \times 10^{10}$copies/μL was 10-fold diluted before use. One Step PrimeScript III RT-qPCR Mix was used according to instruction. The standard curve was obtained according to the average threshold cycle (Ct) and the logarithm of the initial copy number.

PBS ZJCX

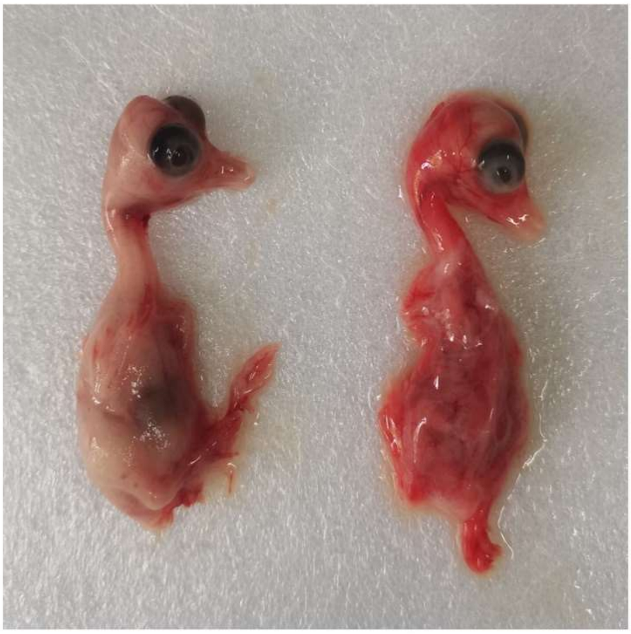

**Figure S2.** Isolation and identification of GAstV in goose embryos. The pathological changes in goose embryos infected with GAstV in the presence of haemorrhage and oedema.

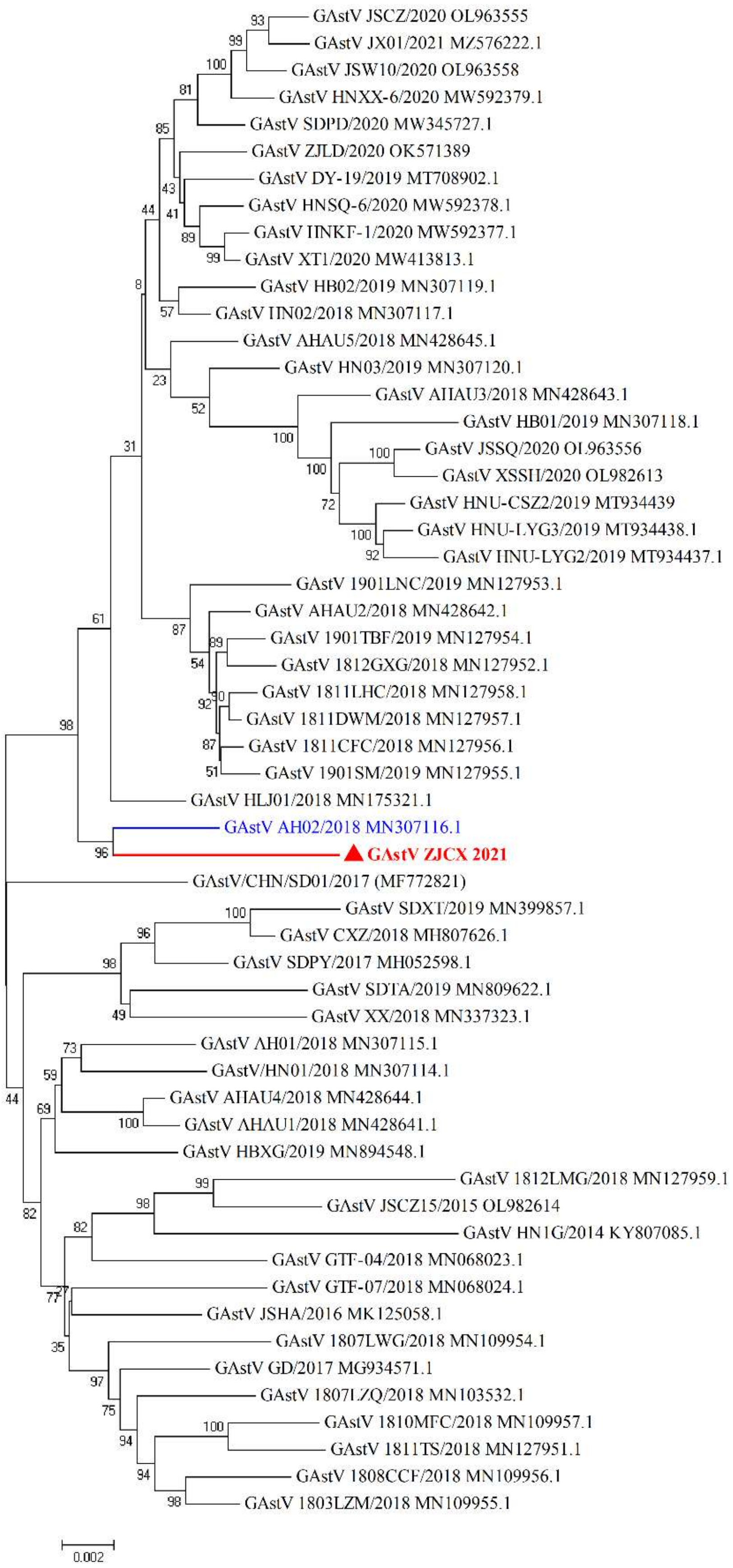


**Figure S3.** Phylogenetic tree of most GAstV II strains in China based on whole-genome sequences. Phylogeny-based genotyping of GAstV strains, constructed based on ClustalW alignment as a neighbor-joining tree via the neighbor-joining method (1,000 bootstrap replicates). The ZJCX (red color) strain get close to AH02 (blue color) in genetic relationships.
